# Adaptive Volume Control in Titanium Alloy for High Temperature Performance

**DOI:** 10.3390/ma12233950

**Published:** 2019-11-28

**Authors:** Pei Li, Xun Sun, Tianlong Zhang, Hualei Zhang, Dong Wang, Qiaoyan Sun, Lin Xiao, Jun Sun

**Affiliations:** 1 State Key Laboratory for Mechanical Behavior of Materials, Xi’an Jiaotong University, Xi’an 710049, China; beckham139@stu.xjtu.edu.cn (P.L.); lxiao@mail.xjtu.edu.cn (L.X.); junsun@mail.xjtu.edu.cn (J.S.); 2 Center of Microstructure Science, Frontier Institute of Science and Technology, Xi’an Jiaotong University, Xi’an 710049, China; xunsun@kth.se (X.S.); zhangtianlong0925@foxmail.com (T.Z.); hualei@xjtu.edu.cn (H.Z.); 3 Applied Materials Physics, Department of Materials Science and Engineering, Royal Institute of Technology, SE-10044 Stockholm, Sweden

**Keywords:** Ti-1023 alloy, adaptive volume, isothermal, high temperature performance

## Abstract

With the increase of time, the shrinkage of materials at fixed temperature could enhance the failure of fasteners. We report a potential way to alter the volume/length of alloy automatically through isothermal aging due to pseudospinodal decomposition mechanism. The volume of Ti-10V-2Fe-3Al alloy first shrunk and then expanded during isothermal aging at 550 °C. It can fit tightly and make up for volume loss. Transmission electron microscopy observation exhibits no obvious coarsening of intragranular α phase with the increasing time. However, composition evolution with time shows a gradual change through energy dispersive spectrometer analysis. The result shows that β stabilizers, V and Fe, are prone to diffuse to the β matrix, while α stabilizers, Al, prefer to segregate to the α phase. First principle calculations suggest that the structure transition for β to α cause the first decrease of volume, and the diffusion of V, Fe and Al is the origin of the later abnormal increase of volume.

## 1. Introduction

Temperature or time dependent volume change of materials is normal in nature and has important influence on applications, e.g., railroad tracks, where temperature changes between summer and winter, day and night, cause big volume changes. The extreme or unusual volume change in material draws a lot of attention based on fundamental and technological standpoint, e.g., thermally operated fasteners and railroad tracks [1]. The failure of fasteners is mainly due to vibration induced loosening with significant wear of the contact surfaces [2,3]. As time increased, a successive shrinkage of the materials was observed exposed at a fixed temperature [4,5,6]. With the increase of time, the shrinkage of materials at fixed temperature could enhance the failure of fasteners because of loosening and wear, which could cause major accident (e.g., China Airlines flight CI-120 in 2007 [7]).

The applications of titanium as a fastener material began in the mid-1960s [8] derived by the reduction of weight of aircraft such as the Airbus A380, which replaced the fastener material with titanium alloy [9,10]. A lot of β-Ti alloys have been used as fastener, such as Ti-5Al-5Mo-5V-3Cr-0.5Fe (wt%, Ti-5553) and Ti-10V-2Fe-3Al (wt%, Ti-1023) [11,12] due to higher strengths than the traditional α-β alloys. The strengthening for titanium alloy is mainly attributed to the precipitation of the α phase. The reason for higher strengths than the traditional α-β alloys is that the α phase in β-Ti alloys can be very fine, undeformable particles with a high volume fraction. Over the years, the need for reliable high-strength titanium fasteners for aircrafts has significantly increased [8]. Besides the strength, the volume change caused by vibration and wearing could also influence the behavior of the fastener, especially at high temperatures [2,3]. It is well known that phase transition could influence the volume due to the volume difference between β and α [13]. Researched has shown that the decrease of the sample length can be measured by using dilatometry during the transformation from β to α; this showed that the volume of the β phase is larger than α [13]. Previous works have shown a gradual decrease of the volume with an increase of time for isothermal aging for β-Ti [4,5]. Whether it is possible a fastener could expand with time and could be knit together more tightly at high temperatures.

The choice of the special temperature for this abnormal thermal expansion is based on pseudospinodal decomposition mechanism [14,15]. The pseudospinodal decomposition mechanism for β to α in Ti-alloys occurs at low temperature [14] and could produce a “two step” process, i.e., the structure change instantaneously and the slow diffusion, which may cause unique properties [16]. To ensure that the pesudospinodal mechanism occurs at certain temperatures, the distance between the alloy composition and C_0_ (the intersection point of the free energy curves of both α and β phases) must be sufficiently small. According to the thermodynamic calculations, the free energy of Ti-1023 at different temperatures is obtained by using V equivalent ([V]) of 11 wt% based on molybdenum equivalents method [16]. As the temperature decreases, C_0_ increases from 5.94% to 10.71%, and the equivalent V composition of Ti-1023 (11%) is close to C_0_ at 550 °C. In addition, to eliminate the effect of ω phase, which appear at low temperature (<500 °C) [17]. We chose 550 °C as the specific temperature to obtain the abnormal phenomenon without the appearance of a ω phase.

In this paper, we report a way to design an adaptive volume control at high temperatures in a Ti-1023 alloy. The present study focuses on the isothermal volume change behavior in a Ti-1023 alloy. It shows the materials with an opposite volume change that first shrink and then expand. The scanning electron microscope (SEM) and transmission electron microscopy (TEM) observations clear show that there is no obvious change for morphology after α has been well precipitated (5 min). The energy dispersive spectrometer (EDS) observations exhibit composition change after structural transition, which suggests that the volume change is obtained by partitioning of solute element. First principle calculations help us to understand the effect of composition change on volume behaviors and suggest that the precipitation of α cause the first decrease of volume, and the diffusion of V, Fe and Al is the origin of later abnormal increase of volume which show gradual expand with time compared to the conventional gradual decrease of the volume with the increase of time.

## 2. Materials and Methods

### 2.1. Experimental Procedure

The as-received material is the hot-rolled Ti-1023 bars. The actual chemical composition was determined by Inductively-Coupled Plasma Atomic Emission Spectroscopy (ICP-AES). The actual chemical composition is 10.2% V, 1.79% Fe, 3.2% Al and the balance Ti (wt.%). The β-transus temperature was 805 ± 5 °C. Specimens for further heat treatment were cut to dimensions φ 16 mm × 10 mm length. The specimens were heat-treated at 1000 °C for 45 min and then directly quenched to 550 °C at a controlled rate of 100 °C/min by Ar gas quenching and annealed for different time. Microstructures were investigated using a JEM-2100F transmission electron microscopy (TEM, JEOL, Tokyo, Japan) operated at 200 kV coupled with energy dispersive spectrometer (EDS), JEM-3010 transmission electron microscopy and SEM-SU6600 scanning electron microscopy (Hitachi, Tokyo, Japan). The length change of cylindrical specimens during heat-treatment was recorded by a Bähr DIL 805 A/D high-resolution differential dilatometer (Baehr-Thermo, Hüllhorst, Germany). The samples for linear thermal expansion tests were mechanically ground by abrasive paper to remove the oxides in surface.

### 2.2. First Principles Calculations

In order to explain the dilatometry, the lattice constants calculations of alloys are based on density functional theory (DFT) [18] and have been performed by means of the exact muffin-tin orbitals method (EMTO) [19]. The volume in alloy can be deduced by averaging the atomic volume for both phases with the atomic ratio of volume fractions (*f*_α_ and *f*_β_), i.e.:

(1) $$V=f_{\alpha }V_{\alpha }+f_{\beta }V_{\beta }$$

The lattice constant (volume) with time can be calculated through introducing different concentration of Al, V, Fe in Ti-1023 according to their different diffusion rate. The faster diffusing β-stabilizers as Fe and V undergo diffusion from α to β and the slower diffusing α-stabilizer as Al undergoes diffusion from β to α simultaneously [17].

## 3. Results

### 3.1. Dilatometer Measurements

**Figure 1:**
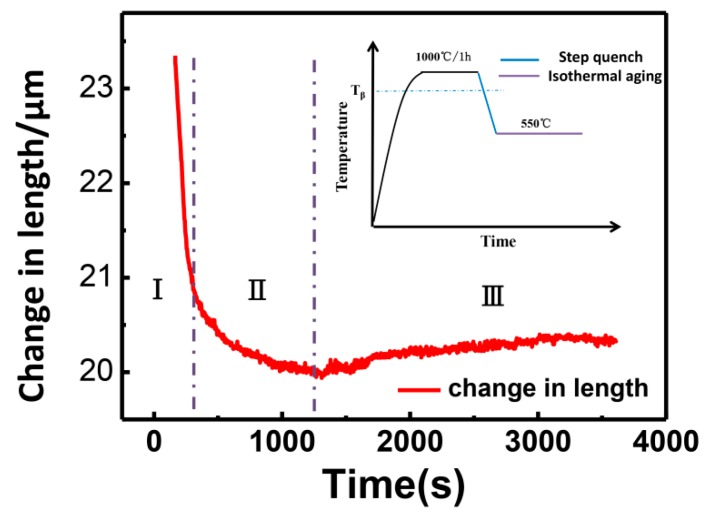
Dilatometric curves of Ti-1023 sample isothermal aging at 550 °C after step quench. Inset picture shows the schematic drawing of step quench process and the purple line shows the aging process.

Figure 1 shows the dilatation curve with isothermal annealing after step quench (the step-quenching was carried out from 1000 °C to a specific temperature at a controlled rate of about 100 °C/min by Ar gas quenching, as shown in the inset of Figure 1). The curve apparently consisted of three stages with two different trends. The first stage (from 165 s to 300 s) shows a sharp linear decrease with almost the same slope and a very large value, which is related to the fast structural transition from β to α [13]. The second stage (from 300 s to 1200 s) shows the decrease of volume, but the value of the slope becomes small, which may be related to the growth process of α precipitates. Previous work shows that a weak growth process of α precipitates exists after rapid nucleation [20]. Interestingly, the volume change in third stage is different from conventional change that decreases with gradual decrease of the slope in previous literatures [4,5,6]. The third stage in our work shows a reversed volume change with time, i.e., the increase of the volume with opposite slope. To our best knowledge, such effect was not reported in literatures.

### 3.2. Change in Structure

**Figure 2:**
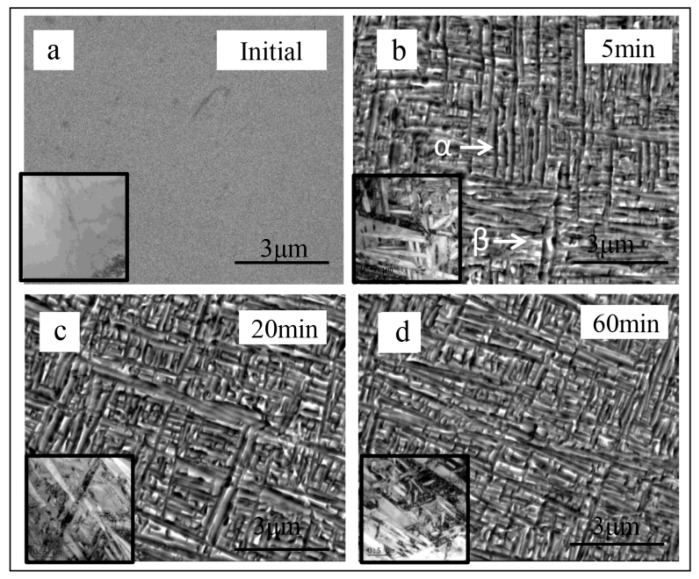
SEM results of Ti-1023 sample after isothermally annealed at 550 °C at different time. SEM image at (**a**) intial time, (**b**) 5 min (**c**) 20 min and (**d**) 60 min. The inset show TEM bright field image.

To understand the abnormal volume change that first shrink and then expand with time of the dilatometric curves in Figure 1, microstructure evolution with different ageing time (at 5 min, 20 min and 60 min) at 550 °C have been exhibited in Figure 2. It shows that there was no obvious coarsening of intragranular α phase with the increase of aging time. Figure 2b–d shows the SEM results of uniformly distribution of α precipitates with high density and the insets show the related TEM results. Figure 2 clearly shows that a complete formed uniform distribution of α precipitation appear at ~5 min and further time increasing could not change the microstructure. Thus, the sharp volume change with large slope in Figure 1 can be attributed to the fast structure change from β to α. However, Figure 2 show no obviously change of the density and size of α precipitates after t = 5 min (there may exists weak growth of α precipitates), which seems inconsistent with the abnormal volume change in Figure 1. The similar microstructure at different time in Figure 2 may suggest that the abnormal volume change (opposite slope) is not related to the structure change. The α phase in the sample are dark on the SEM image, while the β phase are bright on the image in Figure 2. To better analysis the structure change, volume fraction of α phase have been calculated through Image Pro-plus 6.0, which show only about 2.5% increase from 5 min to 60 min. At 5 min, the volume fraction of α phase is 57.1%. At 20 min, the volume fraction of α phase increase 2.4% to 59.5% and the volume fraction of α phase final reach 59.6% at 60 min.

### 3.3. Change in the Composition

**Figure 3:**
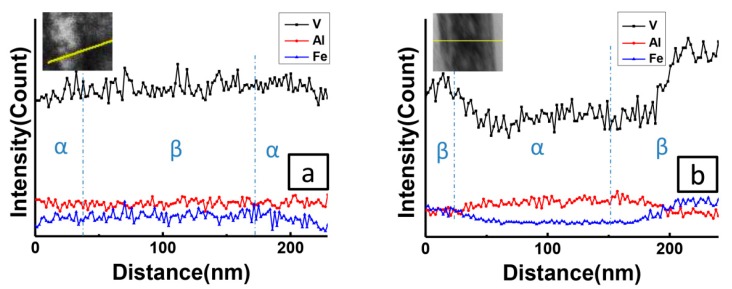
(**a**) The composition profile across the interface of α and β phase after isothermally annealed at 550 °C for 5 min. Inset picture shows the locations in related scanning transmission electron microscopy (STEM) results. (**b**) The composition profile across the interface of α and β phase after isothermally annealed at 550 °C for 60 min. Inset picture shows the locations in related STEM results.

To further understand the abnormal volume change with aging time, the compositional changes are measured by using EDS as shown in Figure 3. The result shows that the compositional redistributions occur during aging, β stabilizers, V and Fe, are prone to diffuse to the β matrix, while α stabilizers, Al, prefer to segregate to the α phase. Surprisingly, there exists no composition difference between two phases at 5 min as shown in Figure 3a, while Figure 2 has shown obvious structure change. This phenomenon reveals that the concentration of α phase after short aging time does not reach equilibrium. After aging for a long time (i.e., 1 h), V, Fe and Al do partition to significant degree and segregation at different phase as indicated in Figure 3b. Therefore, the elements diffusion mainly occurs after 5 min. Combining SEM and TEM results in Figure 2; the abnormal volume change after a long time of aging in Figure 1 could be attributed to the gradual composition diffusion after structure transition. The transformation from β to α involves both a slow elements diffusion and rapid lattice structure change.

## 4. Discussion

**Figure 4:**
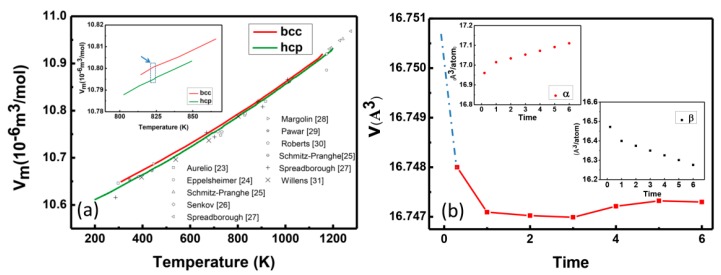
Calculated volume change by Calphad approach and calculated lattice changes and volume change as a function of content by first principles calculations. (**a**) Calculated V_m_ change of Ti for bcc and hcp structure as a function of temperature [22] and compared with the experimental data [23,24,25,26,27,28,29,30,31]. (**b**) Calculated volume change as a function of time. Inset picture shows volume of α and β versus time, respectively.

As usual, resistivity or dilatometry variation is proportional to the amount of α-phase according to Johnson–Mehl–Avrami (JMA) theory [4,20,21]. The β phase is body-centered cubic (bcc) structure and α phase is hexagonal close-packed crystal structure (hcp). The volume of Ti with the bcc and hcp structures as a function of temperature was studied by means of the Calphad approach and related experimental data have been fitted and shown in Figure 4a [22]. The volume per atom of β phase is about 10.800 × 10^−6^ m^3^/mol and the volume per atom of α is about 10.795 × 10^−6^ m^3^/mol. Figure 4a shows that the α phase has smaller lattice/volume than that of β phase, which may result in a decrease of material volume after a short time of aging in stage I in Figure 1 due to β to α phase transition. The dilatation change in stage II is about 4.3% compared to the whole change. The small volume fraction change of α as shown in Figure 2 contributes to the slow decrease of materials volume of stage II in Figure 1.

The followed diffusion process could be the origin of abnormal change of volume with time as shown in stage III of Figure 1. First principles calculations for Ti-1023 with different alloy composition and α volume fraction are shown in Figure 4b. The alloy composition changes according to the diffusion rate based on Figure 3 and α volume fraction based on experimental measurements (see Figure 2) with increasing time. After a sharp linear decrease (by structure transition), there was almost no change of α volume fraction, but composition shows obvious change with time. The increase of concentration of V, Fe and Al elements leads to lattice shrinking of both β and α lattice based on vegard’s law [32]. The β-stabilizers V and Fe undergoes diffusion from α to β, and Al atom are simultaneously diffusing from β to α and then concentrated in α, resulting in the opposite change of α and β lattice as indicted in inset of Figure 4. The opposite effect of lattice changes leads to a shrinking or expanding volume change. First principles calculations reveal the volume change by obvious composition change with increasing time.

The transformation in such a system involves two processes: structure change and long-range diffusion based on the pseudospinodal mechanism [14,15]. Since structure change is much faster than long-range diffusion [33], structure change is the main process in the first stage. At this moment, the α matrix is slightly different from the β matrix in composition. The nucleation rate is extremely high because there is no need for long term diffusion. These dramatic dilatometry drops are believed to be due to the precipitated of α as shown in other work [4,5,21]. The smaller volume per atom of α than that of β at 823 K is shown in Figure 4a (by blue arrows). The moment that α is well precipitated, the composition is still far away from equilibrium. The subsequent aging process will make the composition diffuse. The time-dependent diffusion-induced composition change could produce competition and lead to a volume dip and peak as shown in Figure 4b.

## 5. Conclusions

Thermal expansion behavior of Ti-10V-2Fe-3Al alloy was investigated during isothermal ageing at 550 °C. The following conclusions can be drawn from the investigations:

The specimens were heat-treated at 1000 °C for 45 min and then directly quenched to 550 °C at a controlled rate of 100 °C/min by Ar gas quenching. The initial structure is fully β phase after step quenching. Then, isothermal annealing is performed at 550 °C; an erratic curve that first shrinks and then expands was observed. The result shows that a complete formed uniform distribution of α precipitation appears at 5 min, and as time progresses, the volume friction increases from 57.1% to 59.6%. After 5 min, there was almost no obvious change of α volume fraction, but the composition shows an obvious change with time. The α precipitated through an pseudospinodal mechanism, which simultaneously involves slow change in the composition and a rapid β to α structure change. The structure transition for β to α caused the first decrease of volume. The diffusional redistribution of atoms is the mainly reason for abnormal expansion effect based on first principles calculations.
